# 

**Published:** 1882-06

**Authors:** 


### *TABLE OF CONTENTS FOR JUNE, 1882.*

ORIGINAL COMMUNICATIONS. PAGE

ART. I.—Obstructions in the Larynx and Trachea. By E. FLETCHER INGALS, A.M., M.D. .... 361
ART. II.—The Alcoholic Appetite. By ROGER MARVIN GRISWOLD, M.D. .... 366

CLINICAL REPORTS.

1. Cancer of the Left Mammary Gland, followed by Tetanus. 2. Cancer of the Antrum. By CHARLES B. ZIEGLER, M.D .... 375
CORRESPONDENCE .... 378

ORIGINAL TRANSLATIONS AND ABSTRACTS.

On Diabetic and Nephritic Neuralgias .... 379
Treatment of Catarrh of the Antrum .... 380
Combination of Herpes Zoster and Muscular Atrophy .... 381
The Influence of the Nervous System upon Pathological Changes in the Skin .... 382
Intracranial Disease and Choked Disc .... 383
The Treatment of Inflammation of the Eustachian Tube .... 383
The Action of Ammonia on the Teeth .... 385
The Epithelia of the Kidney .... 389

SELECTIONS.

The Tubercle Parasite—A New Theory of Consumption .... 389
Vaccinal Skin Eruptions .... 392
Infectivity of Scarlet Fever .... 395
Action of Chloroform .... 396
The Surgery of the Kidney .... 397
Treatment and Cure of Nasal Catarrh .... 398
Some Experiments on the Action of Organic Matters upon Silver Salts .... 399
The Physiological Action of Commercial Aconitines .... 400
The Disorders of Primary Dentition .... 402
Genital Irritation .... 407
Migrations of the Ovum .... 407
Headaches in Children .... 408

EDITORIAL DEPARTMENT.

The Exclusion of the New York Delegates from the American Medical Association .... 409
The Alleged Discovery of the Cause of Tuberculosis .... 409
Some False Impressions Corrected .... 410
The Baltimore College of Dental Surgery .... 410
New York College of Dentistry .... 411
CURRENT NEWS AND OPINION .... 411–414
REVIEWS AND BIBLIOGRAPHICAL NOTICES .... 414–416

POPULAR SCIENCE DEPARTMENT.

Dangerous Properties of Dusts .... 416
Ancient Views Concerning Earthquakes .... 420
Is it Worth Domesticating? .... 420
Electric Horticulture .... 421
The Colorado Desert .... 421
A Curious Problem .... 422
The "Smartness" of Worms and Fish .... 423
Using a Gun Camera .... 424
A New Preservative Compound .... 424
War Handkerchiefs .... 426
A New Theory of the Formation of Coal .... 427
Action of Parasitic Fungi .... 427
The Blue Color of Water .... 428
Virchow on Soups and Broths .... 428
Suicide Statistics .... 430

